# Supplementary material for: A synonymous KCNH2 polymorphism and methadone trough level influence QTc prolongation in Kelantanese Malay recipients of methadone maintenance therapy (MMT) in Malaysia
Source: PLoS One. 2025 May 5;20(5):e0322724. doi: 10.1371/journal.pone.0322724 (PMC12052182; doi:10.1371/journal.pone.0322724)
Supplement: S1 Table — (DOCX) [file pone.0322724.s001.docx]

|  | **Sequence** | **GC content (%)** | **Calculated T_m_ (^o^C)** | **Size of PCR product (bp)** | **Primer concentration (pmol / µL)** | **Vol (µL)/reaction** |
| --- | --- | --- | --- | --- | --- | --- |
| **1^st^ PCR** | | | | | | |
| **Exon 6** |  |  |  |  |  |  |
| *KCNH2* EX6FW | 5′-TGC TGT GGT CGC TTG GCT GAG G- 3′ | 64 | 62 | 741 | 0.2 | 1.0 |
| *KCNH2* EX6REV | 5′-CAG CTG CCT TGC CAC CAT GTC TC-3′ | 61 | 61 |  | 0.2 | 1.0 |
| **Exon 8** |  |  |  |  |  |  |
| *KCNH2* EX8FW | 5′-CAT CGT GAC ATG GTT TGC GGG CT-3′ | 57 | 58 | 670 | 0.15 | 1.0 |
| *KCNH2* EX8REV | 5′-TAG AGA CCA TTC CCG CCC TGG G-3′ | 64 |  |  | 0.15 | 1.0 |
| **Exon 9** |  |  |  |  |  |  |
| *KCNH2* EX9FW | 5′-TGA CAT GGA GGG GTC GGA TGG T-3′ | 59 |  | 578 | 0.25 | 1.0 |
| *KCNH2* EX9REV | 5′-TGG CGG ATC CTG AAG GGA AGG-3′ | 62 |  |  | 0.25 | 1.0 |
| **Exon 11** |  |  |  |  |  |  |
| *KCNH2* EX11FW | 5′-TAG GCT TGC CCT GGA GGG TGG A -3′ | 64 |  | 430 | 0.3 | 1.25 |
| *KCNH2* EX11REV | 5′-TGG GGC GCC CAG CCC TAC TTT T-3′ | 64 |  |  | 0.3 | 1.25 |

|  | **Sequence** | **GC content (%)** | **Calculated T_m_ (^o^C)** | **Size of PCR product (bp)** | **Primer concentration (pmol / µL)** | **Vol (µL)/reaction** |
| --- | --- | --- | --- | --- | --- | --- |
| **2^nd^ PCR**  **Set A** | | | | | | |
| *KCNH2* 2690A>C wt FW | 5′-CTT CCG CAG GCG CAC GGA CAA- 3′ | 67 | 62 | 248 | 0.4 | 0.75 |
| *KCNH2* 2690A>C mt FW | 5′-CTT CCG CAG GCG CAC GGA CAC-3′ | 71 | 63 |  | 0.4 | 0.75 |
| *KCNH2* EX11 RV  (COMMON) | 5′-TCA GTG CTC ACA GAG ACC CCA G-3′ | 79 | 58 |  | 0.3 | 0.75 |
| *KCNH2* 1956C>T wt FW | 5′-CAC GCC CCC AGC CCT CAT GTA T-3′ | 64 | 60 | 478 | 0.25 | 0.75 |
| *KCNH2* 1956C>T mt FW | 5′-CAC GCC CCC AGC CCT CAT GTA C-3′ | 68 | 60 |  | 0.25 | 0.75 |
| *KCNH2* EX8 RV  (COMMON) | 5′-TAG AGA CCA TTC CCG CCC TGG G-3′ | 64 | 59 |  | 0.15 | 0.75 |

|  | **Sequence** | **GC content (%)** | **Calculated T_m_ (^o^C)** | **Size of PCR product (bp)** | **Primer concentration (pmol / µL)** | **Vol (µL)/reaction** |
| --- | --- | --- | --- | --- | --- | --- |
| **Set B** | | | | | | |
| *KCNH2* 2350C>T wt FW | 5′-ACC GCC CTG TAC TTC ATC TCC C-3′ | 59 | 57 | 308 | 0.4 | 1.25 |
| *KCNH2* 2350C>T mt FW | 5′-ACC GCC CTG TAC TTC ATC TCC T-3′ | 55 | 57 |  | 0.4 | 1.25 |
| *KCNH2* EX9 RV  (COMMON) | 5′-CTA CTG CCC AGG CTA GAG GAT C-3′ | 59 | 57 |  | 0.25 | 1.25 |
| *KCNH2* 1539C>T wt RV | 5′-ACC TCC TCA GAG CCA GAG CCG-3′ | 67 | 61 | 584 | 0.3 | 0.75 |
| *KCNH2* 1539C>T wt RV | 5′-ACC TCC TCA GAG CCA GAG CC**A** -3′ | 62 | 59 |  | 0.3 | 0.75 |
| *KCNH2* EX6 FW  (COMMON) | 5′-TGC TGT GGT CGC TTG GCT GAC G-3′ | 64 | 62 |  | 0.2 | 0.75 |
