# Supplementary material for: A synonymous KCNH2 polymorphism and methadone trough level influence QTc prolongation in Kelantanese Malay recipients of methadone maintenance therapy (MMT) in Malaysia
Source: PLoS One. 2025 May 5;20(5):e0322724. doi: 10.1371/journal.pone.0322724 (PMC12052182; doi:10.1371/journal.pone.0322724)
Supplement: S1 Fig — (PDF) [file pone.0322724.s002.pdf]

Fig 2. The PCR products for the four KCNH SNPs

(A)The PCR products for the four KCNH SNPs in ten MMT recipients

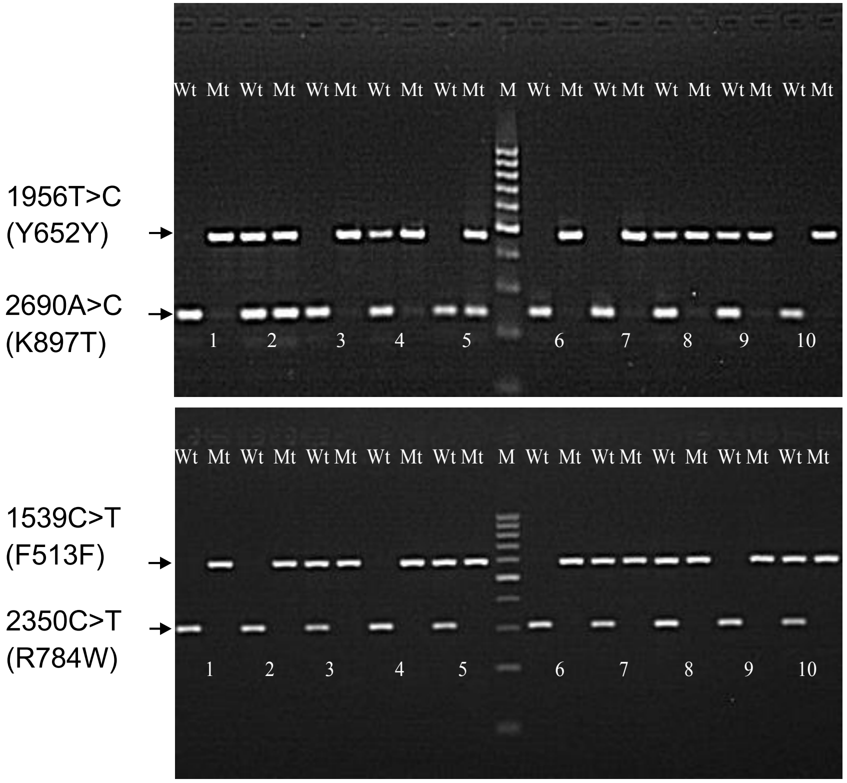

(B) Second PCR Products of Set A

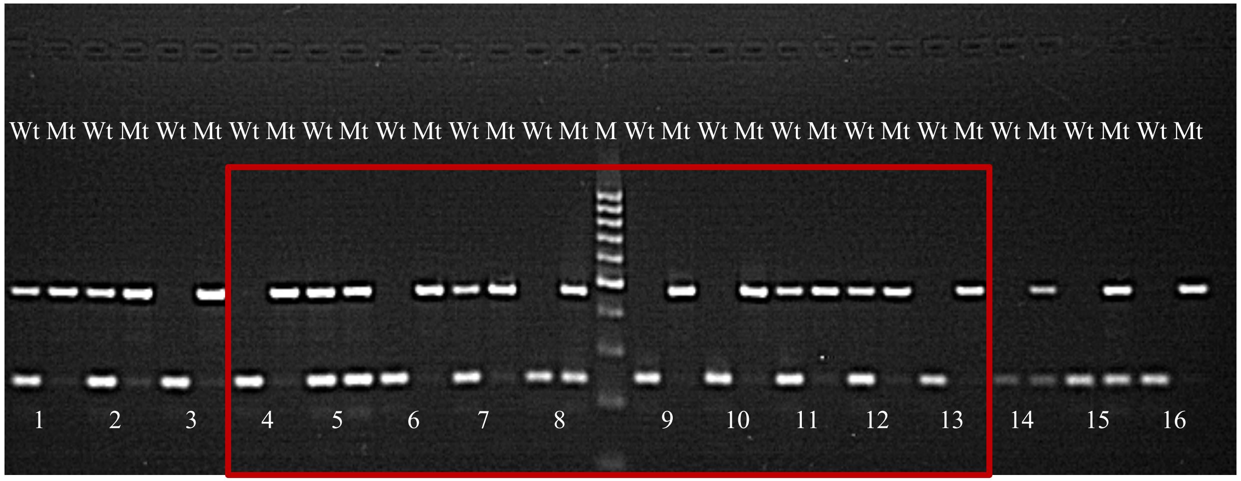

(C) Second PCR Products of Set B

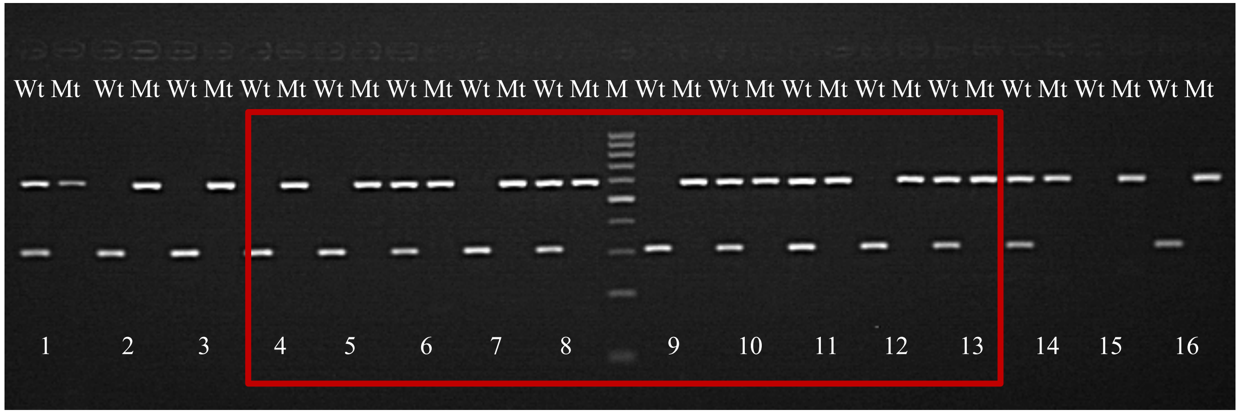

Fig. Agarose gel electrophoresis of PCR amplification. (A) This figure demonstrated the results presented in manuscript (Figure 2). The original imaging data of PCR products of Set A (B) and Set B (C).
