# Supplementary material for: A synonymous KCNH2 polymorphism and methadone trough level influence QTc prolongation in Kelantanese Malay recipients of methadone maintenance therapy (MMT) in Malaysia
Source: PLoS One. 2025 May 5;20(5):e0322724. doi: 10.1371/journal.pone.0322724 (PMC12052182; doi:10.1371/journal.pone.0322724)
Supplement: S2 Fig — (PDF) [file pone.0322724.s003.pdf]

Fig S2. The PCR products for the four KCNH SNPs from an additional 12 representative samples

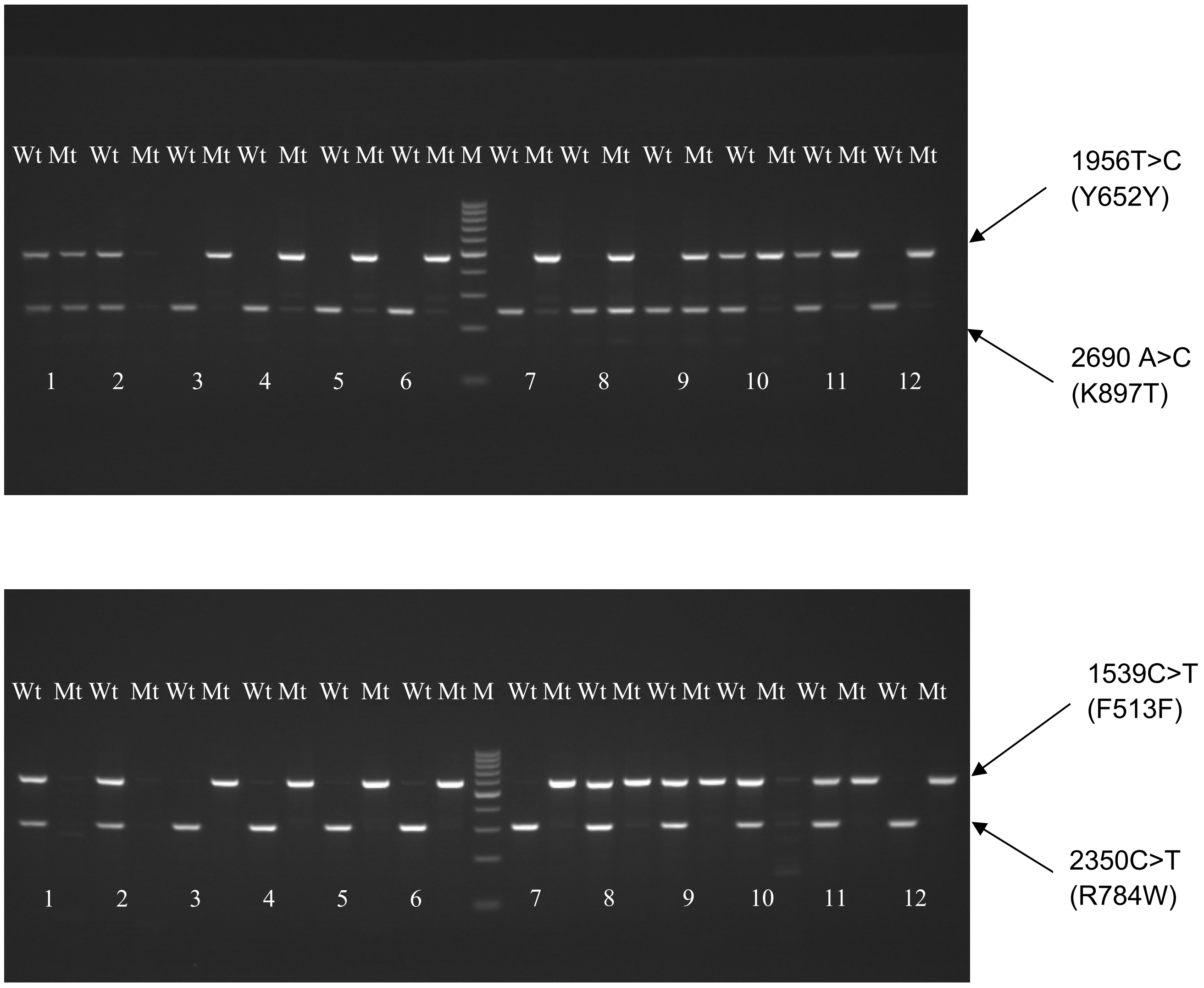

Fig. Agarose gel electrophoresis of PCR amplification. The original imaging data of PCR products of Set A (A) and Set B (B) for additional 12 representative samples.
